# Preclinical characterization of HEC116094, an oral inhibitor of the influenza A virus polymerase PB2 subunit

**DOI:** 10.1080/22221751.2026.2695531

**Published:** 2026-07-10

**Authors:** Longyu Nie, Yunfu Chen, Pu Wang, Yong Xie, Yinglin Zuo, Yingjun Zhang, Baohua Gu, Kai Lin, Jing Li, Lei Zhang

**Affiliations:** a School of Biology and Biological Engineering, South China University of Technology, Guangzhou, People’s Republic of China; b HEC Research and Development Center, Sunshine Lake Pharma Co., Ltd, Dongguan, People’s Republic of China; c State Key Laboratory of Anti-Infective Drug Discovery and Development, Sunshine Lake Pharma Co., Ltd, Dongguan, People’s Republic of China; d South China University of Technology, Guangzhou, People’s Republic of China

**Keywords:** Influenza virus, PB2 inhibitor, VX-787, baloxavir marboxil, drug resistance

## Abstract

Influenza A virus remains a global health threat due to its high mutation rate and its resistance to existing neuraminidase and polymerase inhibitors. The polymerase basic protein 2 (PB2) subunit is a promising target for novel therapeutics due to its critical role in inhibiting viral transcription and replication. Here, we reported the preclinical characterization of HEC116094, a novel PB2 inhibitor, against influenza A virus. HEC116094 exhibited potent *in vitro* activities against multiple laboratory strains (IC_50_: 0.012–0.069 nM) and the highly pathogenic avian influenza strains H5N1 and H7N9 (IC_50_: 0.071–0.122 nM). These activities are more potent than VX-787 (Pimodivir) and oseltamivir. Our studies demonstrated that HEC116094 treatment could reduce the extent of weight loss and maintain a 100% survival rate in BALB/c mice, even when initiated 72 h post-infection. In addition, HEC116094 exerted potent *in vitro* synergy and *in vivo* therapeutic benefits when combined with oseltamivir. Furthermore, HEC116094 displayed minimal kinase inhibition and excellent PK characteristics. Superior preclinical activity demonstrated that HEC116094 was a more potent PB2 inhibitor against influenza A virus than VX-787 in this study. Currently, HEC116094 is under evaluation in Phase I clinical studies.

## Introduction

Influenza virus (IV) is a member of the orthomyxoviridae family, characterized by its segmented genome and single-stranded RNA structure with negative-sense polarity. They are classified into four types (A, B, C, and D), based on the antigenicity of nucleoproteins (NPs) and matrix (M) proteins [1]. Influenza A and B viruses circulate to cause seasonal epidemics. According to World Health Organization (WHO) data, there are approximately a billion cases of seasonal influenza annually, including 3–5 million cases of severe illness and 290,000–650,000 respiratory deaths [2,3].

Vaccination remains a cornerstone of influenza prevention, but its effectiveness varies due to antigenic variability and challenges in predicting circulating strains [4]. Additionally, emerging highly pathogenic avian influenza (HPAI) strains, such as H5N1, pose significant challenges for vaccine development due to their zoonotic potential and high mortality rates [5]. Current antiviral therapies, including neuraminidase inhibitors (e.g. oseltamivir), polymerase inhibitors (e.g. baloxavir marboxil), and M2 ion channel blockers (e.g. amantadine), have reduced influenza morbidity and mortality [6,7]. However, their efficacy is limited by the increasing drug resistance, suboptimal pharmacokinetics, and reduced effectiveness in late-stage infections. M2 inhibitors like amantadine are no longer recommended due to widespread drug resistance. Furthermore, oseltamivir-resistant H1N1 strains with mutations in neuraminidase (NA) and baloxavir-resistant variants with I38 T/M/F mutations in the polymerase acidic (PA) are spreading which underscores the urgent need for novel antivirals with higher genetic barriers to resistance [8].

The RNA-dependent RNA polymerase (RdRp) of the influenza virus is composed of three subunits: a polymerase acidic protein (PA), polymerase basic protein 1 (PB1), and polymerase basic protein 2 (PB2) subunit [9,10]. Since the viral RdRp lacks capping ability, the influenza virus must orchestrate viral genome transcription and replication through a unique cap-snatching mechanism [11]. In this process, the PB2 subunit first binds to the host RNA polymerase II C-terminal domain (CTD) and then selectively captures host mRNAs containing a cap-1 (m7GpppNm) structure [12,13]. PA endonuclease then cleaves approximately 9–14 nucleotides downstream of the cap, resulting in the generation of primers with the 3’-hydroxyl group [14]. Finally, PB1 coordinates RdRp assembly through its N-terminal oligomerization domain and catalyzes RNA elongation [15]. Current approved antiviral drugs targeting influenza viral RdRp are PB1 substrate analogs (favipiravir) [15]. PB2 is an attractive target for antiviral development because PB2 is essential for viral transcription and replication. It also shows less annual variation than haemagglutinin and neuraminidase. Although drugs targeting PB2 may have advantages in inhibiting influenza virus infection [16,17], development of VX-787, a PB2 inhibitor, has been discontinued, as it failed in the Phase III clinical studies [18–20].

**Figure 1.**
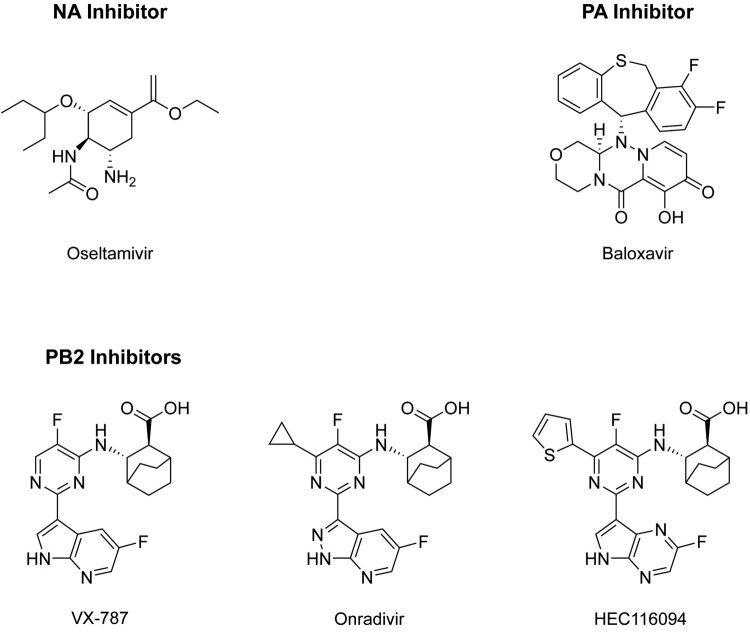
The structure of small molecule drugs for treatment of the influenza virus. The structures of oseltamivir, baloxavir, VX-787, onradivir, and HEC116094.

Here, we report the preclinical antiviral activity of HEC116094, a novel oral PB2 inhibitor for influenza A virus. HEC116094 is optimized based on the structure of VX-787 (Figure 1) [16,21]. Our data demonstrate its potent activities against laboratory and highly pathogenic avian influenza strains *in vitro* and *in vivo*, with strong synergy when combined with oseltamivir. With greater selectivity than VX-787, HEC116094 may offer a stronger treatment effect.

## Materials and methods

### Ethics statement

All the animals were housed in a pathogen-free barrier facility. All animal experiments in this study were in accordance with protocols and procedures approved by the Institutional Animal Care and Use Committee of Sunshine Lake Pharma Co., Ltd. The animal care and use protocol was adhered to the Chinese National Laboratory Animal Guidelines for Ethical Review of Animal Welfare.

### Viruses, cell culture, and chemicals

All experiments involving infectious viruses were performed under biosafety level 2 (BSL-2), BSL-3, or animal biosafety level 2 (ABSL-2) conditions. Low pathogenic influenza virus strains were purchased from the America Type Culture Collection (ATCC). Highly pathogenic avian influenza viruses H5N1（A/Chicken/Henan/12/2004）and H7N9（A/Nanjing/8/2013）were provided, managed, and reserved in BSL-3 conditions by Wuhan Institute of Virology, Chinese Academy of Sciences. Drug-resistant influenza viruses, EV-71, RSV, HSV-1, and HBV were provided by WuXi AppTec (Shanghai, China) Co., Ltd. All the influenza virus strains were grown in MDCK cells, and the viral titres were determined by standard plaque assay in MDCK cells [22,23].

MDCK cells were obtained from ATCC and cultured in Dulbecco’s modified Eagle medium containing 1% penicillin–streptomycin and 10% fetal bovine serum at 37°C with 5% CO_2_.

Throughout this article, oseltamivir collectively refers to both oseltamivir phosphate (used *in vivo*) and oseltamivir carboxylate (used *in vitro*). HEC116094, VX-787, oseltamivir, and baloxavir were synthesized at Sunshine Lake Pharma Co., Ltd. AG7088, BMS-433771, Acyclovir, and Entecavir were provided by WuXi AppTec (Shanghai, China) Co., Ltd.

### Compound antiviral activity and cytotoxicity

MDCK cells were seeded at 15,000 cells per well in 96-well cell culture plates and cultured overnight in a 37°C, 5% CO_2_ incubator. The next day, diluted compounds and viruses were added, and the final concentration of DMSO in the cell culture medium was 0.5%. Cells were cultured in a 5% CO_2_, 33°C, or 37°C incubator for 5 days (All virus strains were cultured at 37°C except for influenza virus A/WSN/33 (H1N1), which was cultured at 33°C) until the compound-free virus infected control wells yielded 80-95% CPE. Each well of cell viability was then measured with the CellTiter-Glo reagent. The cytotoxicity test of the compound was carried out in parallel with the antiviral test, and the experimental conditions were consistent with the antiviral test except that there was no viral infection. The antiviral activity and cytotoxicity of the compound are expressed by the inhibitory activity and cytotoxicity, respectively. IC_50_ and CC_50_ values were analyzed using GraphPad Prism software for nonlinear fitting analysis of the inhibitory activity of the compounds.

### In vitro synergy/antagonism experiments

MDCK cells were seeded at 15,000 cells per well in 96-well cell culture plates and cultured overnight in a 37°C, 5% CO_2_ incubator. The next day, HEC116094 and oseltamivir were diluted serially in 2-fold increments by 7 concentration points. The two compounds were then orthogonally matched at 7 different concentrations, and the compounds were added to the 96-well plate for a total of three replicates. Influenza virus A/PR/8/34 (H1N1) was subsequently added. The final concentration of DMSO in the cell culture medium was 0.5%. Cells were incubated at 37 °C in a 5% CO_2_ incubator for 5 days. Cell viability was detected using the CCK8 reagent, and the raw data were used for compound antiviral activity and cytotoxicity calculations. The experimental data were processed by MacSynegy software [24], and the combined efficacy of HEC116094 and oseltamivir against influenza virus A/PR/8/34 (H1N1) was analyzed.

### Plaque reduction assay

MDCK cells were seeded at a density of 500,000 cells per well in 6-well cell culture plates and cultured overnight in a 37°C, 5% CO_2_ incubator. The next day, compounds and viruses (60 PFU per well) were added, and the compounds were tested for 5 concentrations in double replicate wells. Set up a virus control (no compound-treated cells infected with virus). The final concentration of DMSO in the cell culture medium was 0.5%. After 2 h of viral infection, the supernatant was aspirated, and a low-melting agarose culture containing compounds of different concentrations was added. Three days later, cells were fixed with 4% paraformaldehyde and stained with crystal violet. Plaque data were used for compound antiviral activity calculations. The antiviral activity of the compound was indicated by the rate (%) of the compound's inhibition of the virus-induced plaques.

### In vitro drug resistance evaluation and assay

MDCK cells were seeded at a density of 500,000 cells per well in 6-well cell culture plates and cultured overnight in a 37°C, 5% CO_2_ incubator. Influenza virus A/WSN/33 (H1N1) was added the next day (MOI = 0.005-0.05). After 2 h of infection, the supernatant was aspirated and a culture containing HEC116094 was added. Set up cell controls (cells, no viral infection or compound treatment) and viral controls (cells infected with virus, no compound treatment). The final concentration of DMSO in the cell culture medium was 0.5%. Compound and cells were cultured at 37 °C, 5% CO_2_ incubator for 1–5 days until virus control or compound treated wells yielded 80-95% CPE, and the supernatant was collected and stored in aliquots at −80°C. Viral titres were determined using plaque assays and used for the next round of screening. HEC116094 was started at a concentration of 0.036 nM. The concentration of HEC116094 used in each round of screening was 2 times that of the previous round.

The sensitivity of influenza virus to HEC116094 was determined by plaque reduction assay. Influenza virus RNA was extracted and reverse transcribed into cDNA by primer (5′-AGCGAAAGCAGG-3′) *in vitro*. The PB2 genes were amplified by PCR (Forward: 5′-ATGGAAAGAATAAAAGAACTAAGGAATCT-3′ and Reverse: 5′-CTAATTGATGGCCATCCGAAT-3′). The PCR products were visualized using electrophoresis, purified by Gel DNA Extraction Kit, and sequenced.

### Mouse infection experiments

For compound *in vivo* antiviral activity research, SPF male BALB/c mice (n = 8, each group) were anesthetized by intraperitoneal injection of Zoleti 50, and then inoculated with the 5000 PFU influenza virus strains A/WSN/33 (H1N1) by nasal drop at day 0. Mice were orally treated with compounds or solvents twice a day in a volume of 10 mL/kg, and the first dose was 4 h prior to infection, or 24, 48, and 72 h after infection for 7 consecutive days. Following infection, mouse body weight and survival were monitored daily for 14 days. Mice that lost more than 35% of their initial body weight or displayed severe symptoms were considered deceased and were euthanized.

For PK/PD characterization of HEC116094, mouse body weight and survival were monitored daily for 5 days following infection. On day 4 post infection, mice blood samples in the HEC116094 groups were collected after 0.25, 0.5, 1.0, 2.0, 4.0, 8.0, 12.0, 12.5, and 24.0 h after the first dose. Blood samples were anticoagulated with K_2_EDTA and centrifuged at 4°C, 7000 g for 10 min to separate plasma. Plasma was inactivated with precipitant and stored in a −80°C freezer until subsequent analysis. The drug concentrations in plasma samples were analyzed by LC-MS. On day 5, mouse lung tissues in each group were collected for detection of viral titres.

### Statistical analysis

Statistical analysis was performed using GraphPad Prism 9.0 analytical software (GraphPad, San Diego, CA, USA). The data were expressed as means ± SD. Viral titre comparisons were analyzed by one-way analysis of variance with vehicle group. Multiplicity adjustment was performed using Dunnett test for post-hoc comparisons. Mice survival was analyzed by Kaplan-Meier method with Log-rank test. *, *P* < 0.05; **, *P* < 0.01; ***, *P* < 0.001. The specific details of the statistical tests were described in the figure legends.

## Results

### In vitro activity of HEC116094 against influenza A virus polymerase and multiple laboratory influenza virus strains

Influenza A virus polymerase reporter system was utilized to assess the inhibitory effect of HEC116094 on influenza A virus polymerase. The results indicated that HEC116094 could significantly inhibit influenza A virus polymerase, with a corresponding IC_50_ value of 0.337 ± 0.002 nM and an IC_90_ of 0.395 ± 0.027 nM, which was stronger than those of VX-787 (IC_50_: 2.411 ± 0.947 nM and IC_90_: 3.739 ± 0.321 nM) and oseltamivir (IC_50_: > 100 μM and IC_90_: > 100 μM) (Table 1).

**Table 1.** Inhibitory effects of HEC116094 on influenza A virus polymerase^a^.

Inhibitory activity against IFV A WSN polymerase	HEC116094	VX-787	Oseltamivir
	(nM)	(nM)	(μM)
IC_50_	0.337 ± 0.002	2.411 ± 0.947	> 100^b^
IC_90_	0.395 ± 0.027	3.739 ± 0.321	> 100

^a^ Data shown represent means ± SD of the results of 3 independent biological experiments.

^b^ Oseltamivir was tested at a maximum concentration of 100 μM.

Next, the selectivity of HEC116094 against influenza virus was evaluated in MDCK cells. HEC116094 demonstrated potent activity against influenza A strains (H1N1 and H3N2), with IC_50_ values ranging from 0.012 to 0.069 nM, surpassing VX-787 and oseltamivir (Table 2). Consistent with the specificity of current PB2 inhibitor for influenza A, HEC116094 and VX-787 did not inhibit influenza B virus (IC_50_: > 100 nM). HEC116094 had low cell cytotoxicity with CC_50_ values of 28.907 ± 1.463 μM (33°C) and 16.160 ± 7.047 μM (37°C) in MDCK cells. In addition, HEC116094 exhibited similar cytotoxicity to VX-787 on A549, HEK293, and Jurkat E6 cells (Table 3). Because HEC116094 exhibited almost ten times the antiviral activity against IAV than VX-787, its selectivity index (SI = CC_50_ / IC_50_) was significantly higher than that of VX-787.

**Table 2.** Inhibitory activities of HEC116094 against multiple influenza virus strains.^a^

Virus strains	HEC116094	VX-787	Oseltamivir
	(nM)	(nM)	(μM)
IFV A/WSN/33 (H1N1)	0.027 ± 0.002	0.160 ± 0.027	0.608 ± 0.541
IFV A/PR/8/34 (H1N1)	0.069 ± 0.001	0.477 ± 0.019	0.400 ± 0.040
IFV A/Weiss/43 (H1N1)	0.012 ± 0.005	0.220 ± 0.106	0.285 ± 0.139
IFV A/Mal/302/54 (H1N1)	0.068 ± 0.009	0.166 ± 0.004	0.140 ± 0.059
IFV A/Hong Kong/8/68 (H3N2)	0.022 ± 0.010	0.125 ± 0.041	17.080 ± 6.318
IFV B/Lee/40	> 100^b^	> 100^b^	0.655 ± 0.183

^a^ Data shown represent means ± SD of the results of 3 independent biological experiments.

^b^ HEC116094 and VX-787 were tested at a maximum concentration of 100 nM.

**Table 3.** The cytotoxicity of HEC116094 on multiple cell lines.^a^

Cell	HEC116094	VX-787	Oseltamivir
	(μM)	(μM)	(μM)
MDCK (33°C)	28.907 ± 1.463	15.097 ± 3.972	> 100^b^
MDCK (37°C)	16.160 ± 7.047	22.553 ± 0.674	> 100^b^
A549	141.233 ± 8.652	> 200^c^	> 100^b^
HEK293	178.333 ± 5.036	> 200^c^	> 100^b^
Jurkat E6	83.093 ± 5.763	112.133 ± 3.073	> 100^b^

^a^ Data shown represent means ± SD of the results of 3 independent biological experiments.

^b^ Oseltamivir was tested at a maximum concentration of 100 μM.

^c^ VX-787 was tested at a maximum concentration of 200 μM.

To assess the specificity of the inhibitory activity of HEC116094 against the influenza A virus, the inhibitory activities of HEC116094 against other viruses were evaluated. Positive-strand RNA viruses (human enterovirus 71), negative-strand virus (respiratory syncytial virus), and two DNA viruses (herpes simplex virus type 1 and hepatitis B virus) were chosen. The results indicated that HEC116094 exhibited no inhibitory activity against the viruses mentioned above, with the exception of influenza A virus (Supplementary Table 1).

### In vitro activity of HEC116094 against highly pathogenic avian influenza viruses

**Figure 2.**
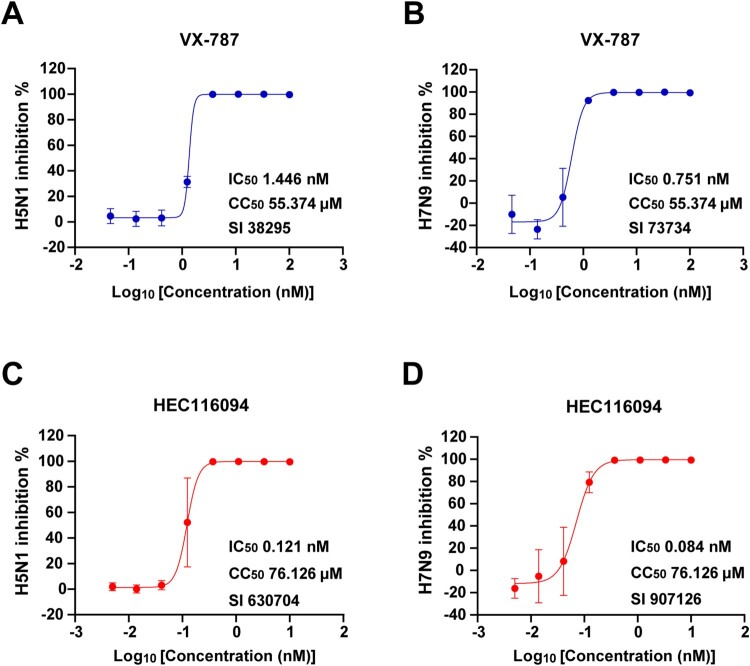
*In vitro* activity of HEC116094 and VX-787 against highly pathogenic avian influenza viruses. (a) Inhibition activity of VX-787 against H5N1. (b) Inhibition activity of VX-787 against H7N9. (c) Inhibition activity of HEC116094 against H5N1. (d) Inhibition activity of HEC116094 against H7N9. IC_50_ values were analyzed using GraphPad Prism software for nonlinear fitting analysis of the inhibitory activity of the compounds. CC_50_, median cytotoxic concentration. SI = antiviral EC_50_/CC_50_. Viability is graphed as percent means ± SD of the results of 3 independent biological experiments.

Highly pathogenic avian influenza viruses can also be transmitted to humans and cause outbreaks [25]. The highly pathogenic avian influenza H5N1 virus clade 2.3.4.4b has caused the death of millions of domestic birds and thousands of wild birds in the USA since January 2022 [5,26]. The antiviral activity of HEC116094 and VX-787 against highly pathogenic avian influenza viruses A/Chicken/Henan/12/2004 (H5N1) and A/Nanjing/8/2013 (H7N9) was assessed on MDCK cells. HEC116094 demonstrated IC_50_ values of 0.121 nM against H5N1 and 0.084 nM against H7N9, both of which were substantially lower than those observed for VX-787, underscoring its enhanced potency against HPAI strains compared to VX-787 (Figure 2).

### Resistance studies of HEC116094 in vitro

**Figure 3.**
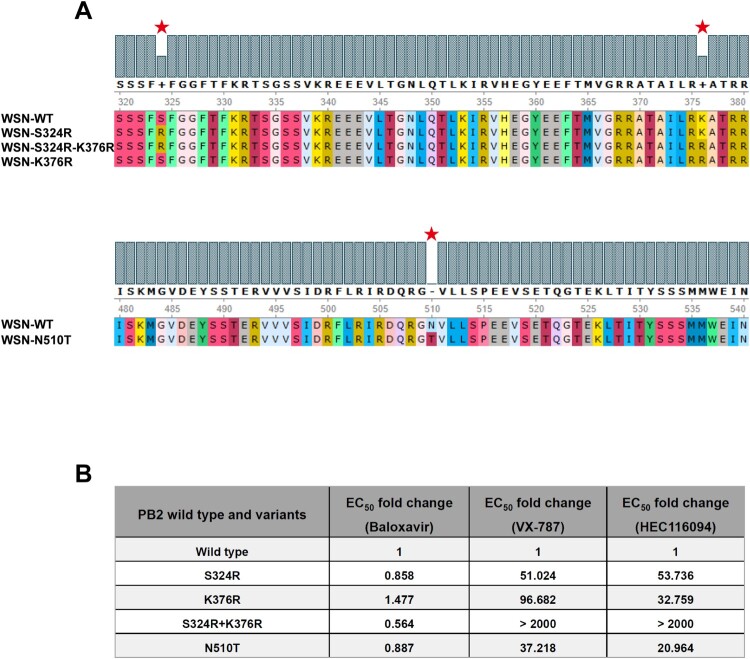
Isolation and characterization of A/WSN/33 (H1N1) variants with reduced HEC116094 susceptibility. (a) PB2 gene sequence alignments of wild-type WSN (WSN-WT) and the resistant WSN viruses by Clustal Omega software and UGENE software. (b) Mean EC_50_ fold changes of baloxavir, VX-787, and HEC116094 against the resistant WSN viruses. Data were assessed with 3 independent biological experiments.

IV A/WSN/33 (H1N1) was passaged over 10 passages in the presence of increasing concentrations of HEC116094 with multiple biological replicates. The results of virus genome sequencing showed that there were four kinds of mutations in the PB2 gene of drug-resistance virus strains (Figure 3(a) and Supplementary note1-5). These resistant influenza viruses showed a significant reduction in sensitivity to VX-787 and HEC116094, while remaining sensitive to baloxavir, as in the wild type (Figure 3(b)). These mutations are key resistance sites against VX-787, and relevant mutant viruses show low replicative ability according to published reports [16,27].

Next, we tested the inhibitory activity of HEC116094 against oseltamivir-resistant A/Weiss/43 (H1N1) influenza viruses, VX-787-resistant A/PR/8/34 (H1N1) influenza viruses, and baloxavir-resistant A/PR/8/34 (H1N1) influenza viruses. The inhibitory activities of HEC116094 against the tested oseltamivir-resistant mutant virus strain and baloxavir-resistant mutant virus strain were comparable to those of the wild type, indicating that HEC116094 had no cross-resistance to oseltamivir and baloxavir. Similar to VX-787, HEC116094 was much weaker than the wild-type virus strain against the tested VX-787 drug-resistant mutant virus strains (Table 4). In conclusion, these results indicated that HEC116094 had cross-resistance with VX-787 and could induce similar drug resistance mutations in the PB2 gene.

**Table 4.** Inhibitory activity of HEC116094 against VX-787-resistant, baloxavir-resistant, and oseltamivir-resistant influenza viruses.^a^

Virus strains	HEC116094 (nM)	VX-787 (nM)	Oseltamivir (μM)	Baloxavir (nM)
VX-787-resistant IFV A/ PR/8/34 (H1N1)	12.000 ± 1.000	39.000 ± 1.000	0.340 ± 0.035	ND^c^
Baloxavir-resistant IFV A/ PR/8/34 (H1N1)	0.048 ± 0.010	0.617 ± 0.035	0.565 ± 0.019	76.740 ± 17.450
IFV A/ PR/8/34 (H1N1)	0.069 ± 0.001	0.477 ± 0.019	0.400 ± 0.040	1.550 ± 0.300
Oseltamivir-resistant IFV A/Weiss/43(H1N1)	0.017 ± 0.007	0.200 ± 0.027	> 100^b^	ND^c^
IFV A/Weiss/43(H1N1)	0.012 ± 0.005	0.220 ± 0.106	0.285 ± 0.139	ND^c^

^a^ Data shown represent means ± SD of the results of 3 independent biological experiments.

^b^ Oseltamivir was tested at a maximum concentration of 100 μM.

^c^ ND means no testing was performed.

### In vivo efficacy of HEC116094 in the mouse influenza A virus infection model

**Figure 4.**
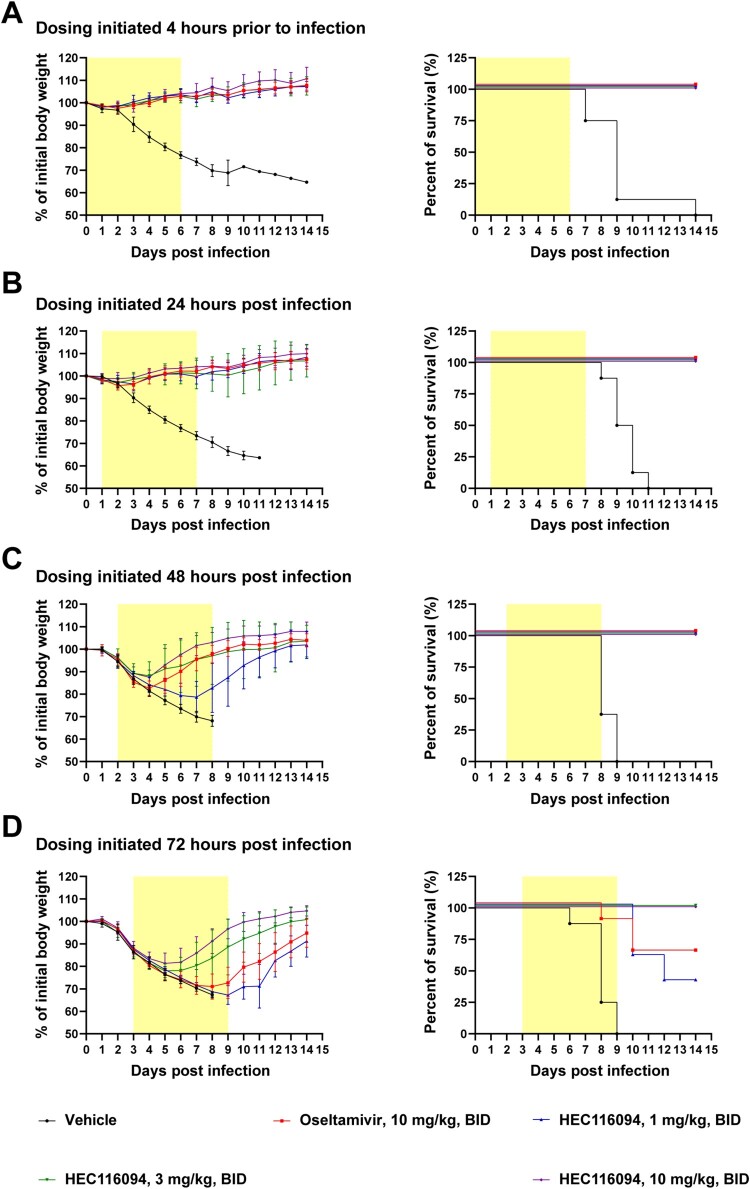
Prophylactic or therapeutic effectiveness of HEC116094 in the mouse influenza A virus infection model. SPF male BALB/c mice (n = 8, each group) were infected with the 5000 PFU influenza virus strains A/WSN/33 (H1N1) followed by administration of vehicle, oseltamivir, or HEC116094 at the indicated doses and start times. The 14-day survival rate and weight are shown. Mice that lost more than 35% of their initial body weight or displayed severe symptoms were considered deceased and were euthanized. The yellow shaded area represents the treatment period. Viability is graphed as percent means ± SD.

In a prophylactic model, BALB/c mice were orally administered HEC116094 (1, 3, or 10 mg/kg, BID) or oseltamivir (10 mg/kg, BID) starting 4 h before lethal A/WSN/33 (H1N1) challenge. All treated mice maintained 100% survival and minimal body weight (BW) loss, unlike vehicle-treated controls, which lost ∼30% body weight and succumbed to infection (Figure 4(a)).

In a therapeutic model, mice received oral administration of HEC116094 (1, 3, or 10 mg/kg, BID) or oseltamivir (10 mg/kg, BID) for 7 days. The results demonstrated that oseltamivir and HEC116094 treatment within 24-48 h post-infection conferred complete survival protection and reduced mouse BW loss across all dosage groups compared to vehicle controls (Figure 4(b) and (c)). When treatment was delayed to 72 h post-infection, 10 mg/kg oseltamivir-treated mice showed significantly reduced survival (62.5%) (Figure 4(d)), however, mice in the 3 and 10 mg/kg HEC116094 regimens maintained 100% survival. These data demonstrated that HEC116094 had better therapeutic potency than oseltamivir *in vivo*.

### Pharmacokinetic and pharmacodynamics (PK/PD) characterization of HEC116094 in the mouse influenza A virus infection model

**Figure 5.**
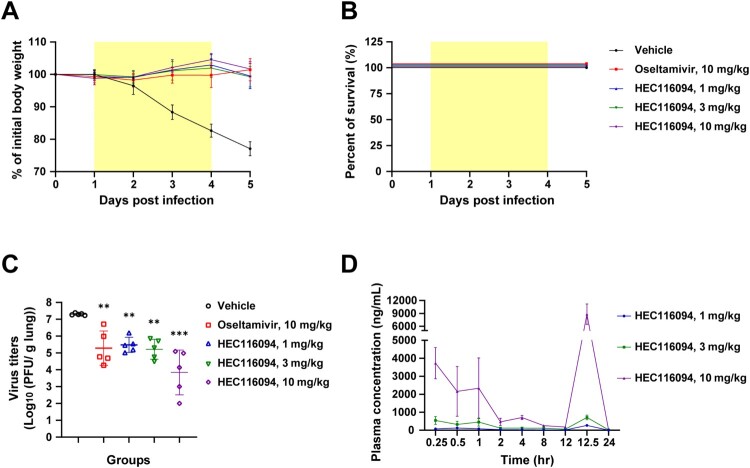
Pharmacokinetic and pharmacodynamics study of HEC116094 in the mouse influenza A virus infection model. SPF male BALB/c mice (n = 5, each group) were infected with the 2500 PFU influenza virus strains A/WSN/33 (H1N1) followed by administration of vehicle, oseltamivir, or HEC116094 twice a day at the indicated doses and start times. The 5-day weight (a) and survival rate (b) are shown. The yellow shaded area represents the treatment period. (c) Viral titres in mouse lungs on day 5. (d) Drug plasma concentration in HEC116094 treated mice. Viability is graphed as percent means ± SD. Viral titre comparisons were analyzed by one-way analysis of variance with vehicle group. Multiplicity adjustment was performed using Dunnett test for all post-hoc comparisons. *, *P* < 0.05; **, *P* < 0.01; ***, *P* < 0.001.

To characterize the PK/PD profile of HEC116094 in the mouse influenza A virus infection model, mice received twice-daily oral doses (1, 3, or 10 mg/kg) for four consecutive days, commencing 24 h post-nonlethal viral challenge. Oseltamivir (10 mg/kg, BID) was used as a control. During the experiment, mouse BW and survival rate were recorded. While vehicle-treated mice exhibited progressive BW loss without mortality, all drug-treated cohorts maintained survival, and they did not lose significant body weight (Figure 5(a) and (b)). After 4-day continuous administration, the viral titres in the lungs of mice in the oseltamivir group (10 mg/kg, BID) and HEC116094 groups (1, 3, and 10 mg/kg, BID) decreased by 2.010 ± 0.457, 1.814 ± 0.202, 2.076 ± 0.265, and 3.464 ± 0.590 log_10_ (PFU/g lung), respectively. There was no statistically significant difference in the viral titres of VX-787 and HEC116094 in the mouse lung compared with oseltamivir. However, in terms of the degree of decline in viral titre in the lungs, the medium dose of HEC116094 (3 mg/kg/dose) was comparable to that of oseltamivir (10 mg/kg/dose). HEC116094 high doses (10 mg/kg/dose) tended to be superior to the same dose of oseltamivir and VX-787 (Figure 5(c) and Supplementary Table 2). In addition, there was a dose dependent increase in plasma HEC116094 concentration in mice (Figure 5(d) and Supplementary Table 2). Compared with the low dose of HEC116094, the high dose of HEC116094 had a better inhibitory effect on the virus, indicating a clear dose–response relationship.

### Combination potential of HEC116094 with oseltamivir in vitro and in vivo

**Figure 6.**
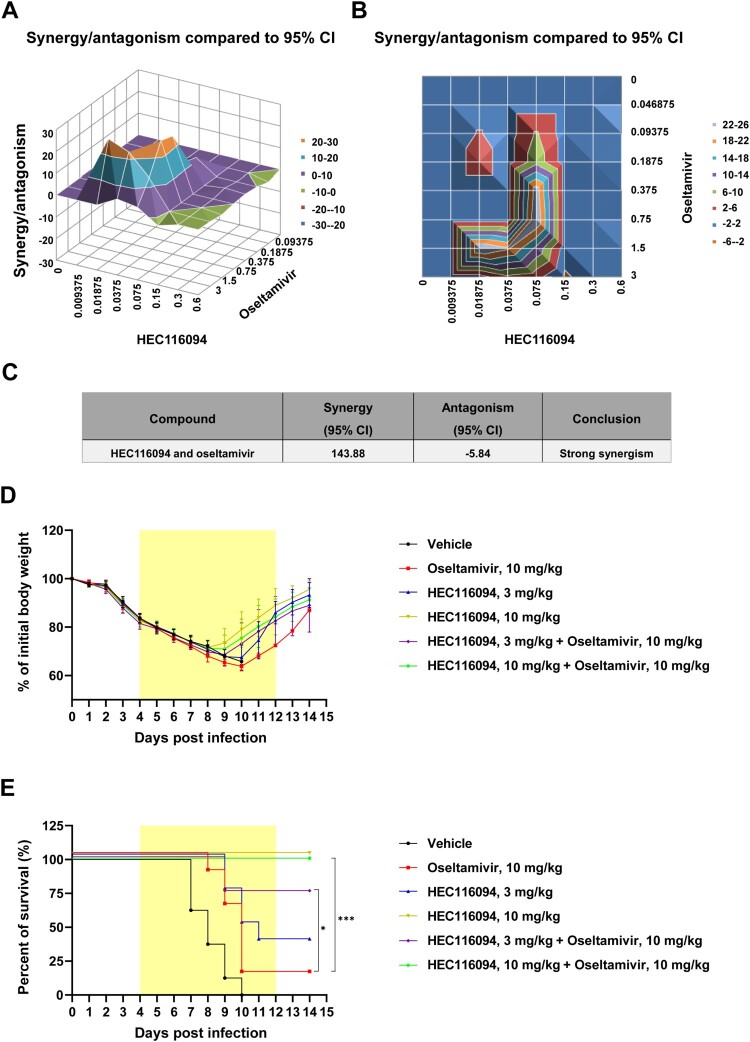
In *vitro* and in *vivo* antiviral combination studies with HEC116094 and oseltamivir (a) Positive and negative synergy diagrams of the combined administration. Data shown were assessed according to the Bliss independence model (95% confidence) with 3 independent biological experiments. Positive values indicate synergy, and negative values indicate antagonism. (b) Top view of Figure 5(a). (c) MacSynergy II Synergy and Antagonism Volumes. MacSynergy II Synergy/Antagonism Volumes Description (95% confidence): < 25 value means insignificant synergism/antagonism; 25–50 value means minor but significant synergism/antagonism; 50–100 value means moderate synergism/antagonism; >100 value means strong synergism/antagonism. SPF male BALB/c mice (n = 8, each group) were infected with the 5000 PFU influenza virus strains A/WSN/33 (H1N1) followed by the administration of vehicle, oseltamivir, or HEC116094 twice a day at the indicated doses and start times. The yellow shaded area represents the treatment period. The 5-day weight (D) and survival rate (E) are shown. Mice that lost more than 35% of their initial body weight or displayed severe symptoms were considered deceased and were euthanized. Viability is graphed as percent means ± SD. Mice survival was analyzed by the Kaplan–Meier method with Log-rank test. *, *P* < 0.05; ***, *P* < 0.001.

Drug resistance is a topic of significant concern in the treatment of infectious diseases caused by rapidly evolving RNA viruses. Combination drug therapy is the standard of care for treating infections caused by rapidly mutating RNA viruses [28]. To evaluate the combined effects of HEC116094 and oseltamivir in *vitro*, an influenza virus cytopathic assay was introduced. The results showed that the synergy and antagonism indices of HEC116094 combined with oseltamivir were 143.88 and −5.84 (95% CI), respectively (Figure 6(a)–(c)). These data indicated that HEC116094 was strongly synergistic with oseltamivir in *vitro*, working through a different mechanism of action.

To assess whether they have synergistic action in *vivo*, male BALB/c mice were treated with the combination drug via oral gavage for nine consecutive days, starting 96 h after a lethal dose of influenza A virus challenge. Compared with the vehicle group, HEC116094 (3 and 10 mg/kg) protected the body weight and improved the survival rate of mice under the set experimental conditions, demonstrating good antiviral efficacy in *vivo*. The combined administration of HEC116094 (3 mg/kg) and oseltamivir (10 mg/kg) could protect the BW of mice and improve the survival rate from 12.5% to 75%, compared with the oseltamivir (10 mg/kg) monotherapy group. In addition, the combined administration of HEC116094 (10 mg/kg) and oseltamivir (10 mg/kg) could protect the body weight of mice and improve the survival rate from 12.5% to 100%, compared with the oseltamivir (10 mg/kg) monotherapy group (Figure 6(d, e)). The results showed that compared with oseltamivir monotherapy, combination with HEC116094 significantly improved survival, indicating a good therapeutic benefit.

## Discussion

Influenza viruses continue to pose a significant health and economic burden to human beings, as they can infect human airways and cause seasonal epidemics and cyclical pandemics [7]. Clinical trial data have shown that both baloxavir marboxil and oseltamivir demonstrate excellent antiviral effects, with the time to alleviation of symptoms being similar for both baloxavir marboxil and oseltamivir [8,28]. Despite the high conservation of the NA catalytic site, some viral variants with reduced susceptibility to NAIs have emerged. The most common NA mutation is the H275Y substitution (H275 and H274 in N1 and N2 numbering, respectively), which confers resistance to both oseltamivir and peramivir [6]. Consistent with a low genetic barrier to resistance, PA I38X variant viruses are almost exclusively isolated from infected patients after exposure to the baloxavir marboxil as early as 24 h post-treatment initiation. Clinical data suggest that resistance may emerge more frequently during the baloxavir marboxil treatment of H3N2 infections [8]. The outcome of a small paediatric study conducted in Japan during the 2016–2017 season showed that the rate of baloxavir resistance was 19.5%, and the majority of patients were treated for illness due to H3N2 [28,29]. Besides the drug resistance, these agents are recommended to be taken within 48 h of the onset of flu symptoms, which limits the treatment window. Hence, new anti-influenza drugs are needed to improve drug resistance and expand the treatment window of the currently approved drugs.

Although VX-787 showed a good preclinical antiviral effect, and combination with or without oseltamivir resulted in significant virological improvements over placebo in a Phase IIb study, there were no statistically significant differences in the time to resolution of influenza symptoms between the VX-787 treatment groups and the placebo group [18]. In the following Phase III clinical studies, VX-787 in combination with standard of care (SoC) showed no additional clinical benefit compared to SoC treatment alone in hospitalized patients [20]. VX-787 was discontinued in 2020 due to futility being observed in the clinical trial. Another PB2 inhibitor, ZSP1273, exhibited better antiviral activity and PK character in the preclinical study compared to VX-787 [30]. In Phase II and III clinical studies, the antiviral activity of ZSP1273 outperformed that of both the placebo and oseltamivir, quickly decreasing viral shedding and shortening the measurable time of viral presence. Additionally, ZSP1273 exhibited similar therapeutic effects in alleviating influenza symptoms compared to oseltamivir [31,32]. ZSP1273 was approved by the National Medical Products Administration (NMPA) for use in adults with IAV infection in May 2025.

Our results demonstrated that HEC116094 exhibits excellent antiviral activities *in vitro* and *in vivo*. In SD rats and Beagle dogs, single-dose HEC116094 exhibited low CL and long T_1/2_ (Supplementary Tables 3 and 4). In addition, HEC116094 did not have mitochondrial toxicity and displayed minimal kinase inhibition (Supplementary Tables 5 and 6). The predominant SI and PK characteristics of HEC116094 in this study indicate that it may have a better treatment outcome against seasonal and highly pathogenic avian influenza viruses than VX-787.

We note that there are some limitations to this study. (i) ZSP1273 exhibited excellent therapeutic effects in clinical trials and was approved in 2025. However, we did not conduct a head-to-head study of HEC116094 and ZSP1273 in this research. The relevant research will be conducted in the following work. (ii) The study of HEC116094s resistance barrier is not systematic. Next-Generation Sequencing (NGS), replication fitness analyses, and *in vivo* resistance studies of HEC116094 could provide us with additional data to assess the resistance barriers. (iii) While delayed treatment at 72 h post-infection achieved 100% survival in BALB/c mice, further virological and immunopathological studies are still needed to verify their potential for an extended therapeutic window.(iv) Q591 K, E627 K, and D701N mutations on PB2 are the pathogenic markers in PB2 of HPAI [33]. Our results showed that HEC116094 exhibited excellent activities against H7N9 (Q591 K mutation on PB2). However, the effects of other mutations on drug resistance have not been assessed. (v) In the influenza B virus (IBV), Gln325 replaces Phe323, thereby eliminating the strong π stacking of pyrimidine rings in VX-787, which greatly weakens VX-787’s activity against the IBV [34]. Therefore, this structure–activity relationship should be considered in subsequent compound designs to improve activity against IBV.

In conclusion, our study indicated that HEC116094 is a highly efficacious and safe inhibitor of influenza A virus PB2 *in vitro* and *in vivo*. Currently, HEC116094 is under evaluation in Phase I clinical studies.

## Supplementary Material

Supplementary material 03.docx
