# Supplementary material for: Preclinical characterization of HEC116094, an oral inhibitor of the influenza A virus polymerase PB2 subunit
Source: Emerg Microbes Infect. 2026 Jul 10;15(1):2695531. doi: 10.1080/22221751.2026.2695531 (PMC13360517; doi:10.1080/22221751.2026.2695531)
Supplement: Supplementary material 03.docx [file TEMI_A_2695531_SM8368.docx]

**Supplementary material**

**Preclinical characterization of HEC116094, an oral inhibitor of the influenza A virus polymerase PB2 subunit**

**Pharmacokinetic study of single-dose HEC116094 given by oral or intravenous administration in SD rats and Beagle dogs**

Male and female SD rats were orally gavaged and intravenously injected with HEC116094 (0.50 mg/kg, QD). For IV administration, HEC116094 is narrowly distributed in SD rats, and the volume of distribution (V_ss_) was 0.29 ± 0.037 L/kg. HEC116094 showed a plasma clearance (CL) of 1.5 ± 0.32 mL/min/kg with a half-life (T_1/2_) of 3.3 ± 0.54 h. After oral administration of HEC116094 in SD rats, HEC116094HCl was rapidly absorbed, and the peak time of plasma concentration (T_max_) was 0.83-1.5 h, and the T_1/2_ was 3.1-3.9 h. After IV and oral administration of HEC116094 in male and female SD rats (0.50 mg/kg, QD), the C_max_ ratio was basically between 0.8-1.2 and AUC_last_ was 0.6-1.3 in male and female SD rats, indicating that there was no significant difference in the absorption of HEC116094 between male and female SD rats. The absolute bioavailability (F) of HEC116094 in fasting and feeding SD rats was 63% and 65%, respectively. The peak concentration (C_max_) ratios between female and male SD rats fed to fasting group were 1.7 and 1.0, and the AUC from time zero to time of last measurable concentration (AUC_last_) ratios were 1.3 and 0.82, respectively, indicating that the effect of food may not be obvious (Supplementary Table 2).

Male and female Beagle dogs were orally gavaged and intravenously injected with HEC116094 (10 mg/kg, QD). For IV administration, HEC116094HCl showed a CL of 11 ± 1.6 mL/min/kg with a T_1/2_ of 3.9 ± 1.7 h, and its V_ss_ was 0.43 ± 0.075 L/kg. After oral administration of HEC116094 in Beagle dogs, HEC116094HCl was rapidly absorbed, and the T_max_ was 0.17-2.5 h. The absolute bioavailability (F) of HEC116094 in fasting Beagle dogs was 22%. In IV group and oral group, the male-to-female ratios of C_max_ were 1.1 and 0.67, and the ratios of AUC_last_ was 1.3 and 0.94, respectively, indicating that there were no obvious differences between males and females Beagle dogs in the feeding group. However, the female-to-male ratios of C_max_ and AUC_last_ were 0.037 and 0.28 in oral feeding group, respectively, which shown that food had a clear effect on the absorption of HEC116094 in female Beagle dog groups (Supplementary Table 3).

**Kinase inhibition and mitochondrial toxicity assays of HEC116094**

Many antiviral drugs work by inhibiting viral enzymes, but some small molecules may interfere with human kinase function due to structural similarities or off-target effects. The inhibitory effects of HEC116094 on 23 human kinases were summarized in Supplementary Table 4. The IC_50_ of HEC116094 against all the human kinases tested was more than 10,000 nM. A study reported that the IC_50_ of VX-787 ranged from 1000 nM to 10,000 nM against GSK3 beta, MAP4K4, MINK, and TNIK, but no significant inhibition of VX-787 against other kinases was observed^1^.In addition, the mitochondrial toxicity of HEC116094 was assessed on the HepG2 cells. Nefazodone and digitonin were used as the positive and negative controls, respectively. The results shown that HEC116094 did not have mitochondrial toxicity (Supplementary Table 5).

These data indicated that HEC116094 displayed minimal kinase inhibition and did not have mitochondrial toxicity.

**Supplementary Table 1. Inhibitory activity of HEC116094 against different viruses.**

| Virus | Cell | Compound | IC_50_ | CC_50_ |
| --- | --- | --- | --- | --- |
|  |  |  | (μM) | (μM) |
| EV71 | RD | HEC116094 | > 10 | > 10 |
|  |  | AG7088 | 0.015 | > 1 |
| RSV | HEp-2 | HEC116094 | > 10 | > 10 |
|  |  | BMS-433771 | 0.030 | > 1 |
| HSV-1 | Vero | HEC116094 | > 10 | > 10 |
|  |  | Acyclovir | 0.785 | > 10 |
| HBV | HepG2.2.15 | HEC116094 | > 10 | > 10 |
|  |  | Entecavir | 0.003 | > 10 |

The maximum tested concentrations of HEC116094, AG7088, BMS-433771, Acyclovir, Entecavir were 10 μM, 1 μM, 1 μM, 10 μM, 10 μM, respectively.

**Supplementary Table 2. PK/PD characterization of HEC116094 in the mouse IAV infection model.**

| Groups | Viral load decline versus vehicle group  Log_10_ (PFU / g lung) | AUC_0-12h_^b^ (ng·h/mL) | AUC_0-24h_^b^  (ng·h/mL) | C_12h_^b^  (ng/mL) | C_24h_^b^  (ng/mL) |
| --- | --- | --- | --- | --- | --- |
| HEC116094  1 mg/kg, BID | 1.814 ± 0.202^a^ | 369 | 2010 | 18 | 3 |
| HEC116094  3 mg/kg, BID | 2.076 ± 0.265^a^ | 1675 | 6074 | 57 | 21 |
| HEC116094  10 mg/kg, BID | 3.464 ± 0.590^a^ | 7685 | 60739 | 175 | 23 |
| Oseltamivir  10 mg/kg, BID | 2.010 ± 0.457^a^ | ND^c^ | ND^c^ | ND^c^ | ND^c^ |

^a^ Data shown represent means ± SD (n = 3, each group).

^b^ Data shown represent means (n = 3, each group).

^c^ ND means no testing was performed.

**Supplementary Table 3. Pharmacokinetic study of single-dose HEC116094 given by oral or intravenous administration in SD rats.**

| **Animal numbers/sex** | 3F | 3M | 3F | 3M | 3F | 3M |
| --- | --- | --- | --- | --- | --- | --- |
| **Food Condition** | Fasted | | Fasted | | Fed | |
| **Formulation** | *i.v* | | *p.o* | | *p.o* | |
| **QD Dose (mg/kg)** | 0.5 | | | | | |
| **T_max_ (h)** | / | / | 1.3 | 1.7 | 1.0 | 0.83 |
| **C_max_ (ng/mL)** | / | / | 522 | 629 | 895 | 649 |
| **AUC_0-t_ (ng·h/mL)** | 5070 | 5750 | 2660 | 4150 | 3530 | 3420 |
| **AUC_0-∞_ (ng·h/mL)** | 5310 | 6240 | 5670 | 4610 | 3760 | 3740 |
| **MRT_0-∞_ (h)** | 2.9 | 3.8 | 4.97 | 7.2 | 5.2 | 5.3 |
| **T_1/2_ (h)** | 3 | 3.6 | 2.5 | 4.4 | 4.0 | 3.3 |
| **CL (mL/min/kg)** | 1.6 | 1.3 | / | / | / | / |
| **V_ss_ (L/kg)** | 0.28 | 0.31 | / | / | / | / |
| **F (%)** | / | / | 53 | 74 | 71 | 60 |

**Supplementary Table 4. Pharmacokinetic study of single-dose HEC116094 given by oral or intravenous administration in Beagle dogs.**

| **Animal numbers/sex** | 3F | 3M | 3F | 3M | 3F | 3M |
| --- | --- | --- | --- | --- | --- | --- |
| **Food Condition** | Fasted | | Fasted | | Fed | |
| **Formulation** | *i.v* | | *p.o* | | *p.o* | |
| **QD Dose (mg/kg)** | 10 | | | | | |
| **T_max_ (h)** | / | / | 0.83 | 0.50 | 1.7 | 0.31 |
| **C_max_ (ng/mL)** | / | / | 2900 | 4360 | 272 | 7440 |
| **AUC_0-t_ (ng·h/mL)** | 17400 | 13800 | 3250 | 3440 | 1000 | 3530 |
| **AUC_0-∞_ (ng·h/mL)** | 17000 | 14000 | 3300 | 3500 | 1100 | 3500 |
| **MRT_0-∞_ (h)** | 4.8 | 2.9 | 2.1 | 2.3 | 5.0 | 1.0 |
| **T_1/2_ (h)** | 0.72 | 0.61 | 2.9 | 3.4 | 3.0 | 2.0 |
| **CL (mL/min/kg)** | 9.6 | 12 | / | / | / | / |
| **V_ss_ (L/kg)** | 0.41 | 0.45 | / | / | / | / |
| **F (%)** | / | / | 19 | 26 | / | / |

**Supplementary Table 5. Summary of the IC_50_ of HEC116094 against 23 kinases by kinase profiling^a^.**

| Kinase | IC_50_（nM） | | |
| --- | --- | --- | --- |
|  | HEC116094 | VX-787^b^ | Staurosporine^b^ |
| ABL1 | > 10000 | > 10000 | 171 |
| CAMK2 alpha | > 10000 | > 10000 | 1 |
| CDK2 | > 10000 | > 10000 | 4 |
| CHK1 | > 10000 | > 10000 | 6 |
| CK1 alpha | > 10000 | > 10000 | > 10000 |
| DYRK3 | > 10000 | > 10000 | 165 |
| EPHA2 | > 10000 | > 10000 | 675 |
| FGFR1 | > 10000 | > 10000 | 26 |
| GSK3 beta | > 10000 | > 3333 | 60 |
| MAP4K4 | > 10000 | 1782 | 2 |
| JAK2 | > 10000 | > 10000 | 6 |
| JNK1 | > 10000 | > 10000 | 940 |
| KDR | > 10000 | > 10000 | 37 |
| LCK | > 10000 | > 10000 | 15 |
| MAPKAPK2 | > 10000 | > 10000 | 318 |
| MINK | > 10000 | > 3333 | 1 |
| MST4 | > 10000 | > 10000 | 4 |
| p38 alpha | > 10000 | > 10000 | > 10000 |
| PDK1 | > 10000 | > 10000 | 5 |
| PKA | > 10000 | > 10000 | 12 |
| SRC | > 10000 | > 10000 | 42 |
| TAOK2 | > 10000 | > 10000 | 18 |
| TNIK | > 10000 | > 3333 | 13 |

Kinase activity was measured by Z’-LYTE® technology (Invitrogen) based on the principle of fluorescence resonance energy transfer (FRET).

^a^ Compounds was tested at a maximum concentration of 10000 nM.

^b^ Data cited from the publicly research article[1].

**Supplementary Table 6. The Mitotoxicity of HEC116094 on HepG2 cell line.**

| Compound | IC_50_ (μM) | | IC_50 (Glucose)_ / IC_50_ _(Galactose)_^a^ |
| --- | --- | --- | --- |
|  | Glucose | Galactose |  |
| Nefazodone | 14.66 | 2.81 | 5.22 |
| Digitonin | 1.97 | 1.05 | 1.87 |
| HEC116094 | 42.12 | 37.52 | 1.12 |

^a^ IC_50 (Glucose)_ / IC_50_ _(Galactose)_ < 3 means no mitotoxicity; 5 > IC_50 (Glucose)_ / IC_50_ _(Galactose)_ ≥ 3 means potent mitotoxicity; IC_50 (Glucose)_ / IC_50_ _(Galactose)_ ≥ 5 means mitotoxicity.

**Supplementary Note 1. WSN-WT PB2 protein sequences:**

MERIKELRNLMSQSRTREILTKTTVDHMAIIKKYTSGRQEKNPALRMKWMMAMKYPITADKRITEMIPERNEQGQTLWSKMNDAGSDRVMVSPLAVTWWNRNGPVTSTVHYPKIYKTYFEKVERLKHGTFGPVHFRNQVKIRRRVDINPGHADLSAKEAQDVIMEVVFPNEVGARILTSESQLTTTKEKKEELQGCKISPLMVAYMLERELVRKTRFLPVAGGTSSVYIEVLHLTQGTCWEQMYTPGGEARNDDVDQSLIIAARNIVRRATVSADPLASLLEMCHSTQIGGIRMVNILRQNPTEEQAVDICKAAMGLRISSSFSFGGFTFKRTSGSSVKREEEVLTGNLQTLKIRVHEGYEEFTMVGRRATAILRKATRRLIQLIVSGRDEQSIAEAIIVAMVFSQEDCMIKAVRGDLNFVNRANQRLNPMHQLLRHFQKDAKVLFQNWGIESIDNVMGMIGILPDMTPSTEMSMRGVRISKMGVDEYSSAEKIVVSIDRFLRVRDQRGNVLLSPEEISETQGTEKLTITYSSSMMWEINGPESVLVNTYQWIIRNWETVKIQWSQNPTMLYNKMEFEPFQSLVPKAVRGQYSGFVRTLFQQMRDVLGTFDTAQIIKLLPFAAAPPKQSRTQFSSLTINVRGSGMRILVRGNSPVFNYNKTTKRLTVLGKDAGPLTEDPDEGTAGVESAVLRGFLILGKEDRRYGPALSINELSNLAKGEKANVLIGQGDVVLVMKRKRNSSILTDSQTATKRIRMAIN*

**Supplementary Note 2. WSN-324R PB2 protein sequences:**

MERIKELRNLMSQSRTREILTKTTVDHMAIIKKYTSGRQEKNPALRMKWMMAMKYPITADKRITEMIPERNEQGQTLWSKMNDAGSDRVMVSPLAVTWWNRNGPVTSTVHYPKIYKTYFEKVERLKHGTFGPVHFRNQVKIRRRVDINPGHADLSAKEAQDVIMEVVFPNEVGARILTSESQLTTTKEKKEELQGCKISPLMVAYMLERELVRKTRFLPVAGGTSSVYIEVLHLTQGTCWEQMYTPGGEARNDDVDQSLIIAARNIVRRATVSADPLASLLEMCHSTQIGGIRMVNILRQNPTEEQAVDICKAAMGLRISSSFRFGGFTFKRTSGSSVKREEEVLTGNLQTLKIRVHEGYEEFTMVGRRATAILRKATRRLIQLIVSGRDEQSIAEAIIVAMVFSQEDCMIKAVRGDLNFVNRANQRLNPMHQLLRHFQKDAKVLFQNWGIESIDNVMGMIGILPDMTPSTEMSMRGVRISKMGVDEYSSAEKIVVSIDRFLRVRDQRGNVLLSPEEISETQGTEKLTITYSSSMMWEINGPESVLVNTYQWIIRNWETVKIQWSQNPTMLYNKMEFEPFQSLVPKAVRGQYSGFVRTLFQQMRDVLGTFDTAQIIKLLPFAAAPPKQSRTQFSSLTINVRGSGMRILVRGNSPVFNYNKTTKRLTVLGKDAGPLTEDPDEGTAGVESAVLRGFLILGKEDRRYGPALSINELSNLAKGEKANVLIGQGDVVLVMKRKRNSSILTDSQTATKRIRMAIN*

**Supplementary Note 3. WSN-376R PB2 protein sequences:**

MERIKELRNLMSQSRTREILTKTTVDHMAIIKKYTSGRQEKNPALRMKWMMAMKYPITADKRITEMIPERNEQGQTLWSKMNDAGSDRVMVSPLAVTWWNRNGPVTSTVHYPKIYKTYFEKVERLKHGTFGPVHFRNQVKIRRRVDINPGHADLSAKEAQDVIMEVVFPNEVGARILTSESQLTTTKEKKEELQGCKISPLMVAYMLERELVRKTRFLPVAGGTSSVYIEVLHLTQGTCWEQMYTPGGEARNDDVDQSLIIAARNIVRRATVSADPLASLLEMCHSTQIGGIRMVNILRQNPTEEQAVDICKAAMGLRISSSFSFGGFTFKRTSGSSVKREEEVLTGNLQTLKIRVHEGYEEFTMVGRRATAILRRATRRLIQLIVSGRDEQSIAEAIIVAMVFSQEDCMIKAVRGDLNFVNRANQRLNPMHQLLRHFQKDAKVLFQNWGIESIDNVMGMIGILPDMTPSTEMSMRGVRISKMGVDEYSSAEKIVVSIDRFLRVRDQRGNVLLSPEEISETQGTEKLTITYSSSMMWEINGPESVLVNTYQWIIRNWETVKIQWSQNPTMLYNKMEFEPFQSLVPKAVRGQYSGFVRTLFQQMRDVLGTFDTAQIIKLLPFAAAPPKQSRTQFSSLTINVRGSGMRILVRGNSPVFNYNKTTKRLTVLGKDAGPLTEDPDEGTAGVESAVLRGFLILGKEDRRYGPALSINELSNLAKGEKANVLIGQGDVVLVMKRKRNSSILTDSQTATKRIRMAIN*

**Supplementary Note 4. WSN-324R-376R PB2 protein sequences:**

MERIKELRNLMSQSRTREILTKTTVDHMAIIKKYTSGRQEKNPALRMKWMMAMKYPITADKRITEMIPERNEQGQTLWSKMNDAGSDRVMVSPLAVTWWNRNGPVTSTVHYPKIYKTYFEKVERLKHGTFGPVHFRNQVKIRRRVDINPGHADLSAKEAQDVIMEVVFPNEVGARILTSESQLTTTKEKKEELQGCKISPLMVAYMLERELVRKTRFLPVAGGTSSVYIEVLHLTQGTCWEQMYTPGGEARNDDVDQSLIIAARNIVRRATVSADPLASLLEMCHSTQIGGIRMVNILRQNPTEEQAVDICKAAMGLRISSSFRFGGFTFKRTSGSSVKREEEVLTGNLQTLKIRVHEGYEEFTMVGRRATAILRRATRRLIQLIVSGRDEQSIAEAIIVAMVFSQEDCMIKAVRGDLNFVNRANQRLNPMHQLLRHFQKDAKVLFQNWGIESIDNVMGMIGILPDMTPSTEMSMRGVRISKMGVDEYSSAEKIVVSIDRFLRVRDQRGNVLLSPEEISETQGTEKLTITYSSSMMWEINGPESVLVNTYQWIIRNWETVKIQWSQNPTMLYNKMEFEPFQSLVPKAVRGQYSGFVRTLFQQMRDVLGTFDTAQIIKLLPFAAAPPKQSRTQFSSLTINVRGSGMRILVRGNSPVFNYNKTTKRLTVLGKDAGPLTEDPDEGTAGVESAVLRGFLILGKEDRRYGPALSINELSNLAKGEKANVLIGQGDVVLVMKRKRNSSILTDSQTATKRIRMAIN*

**Supplementary Note 5. WSN-510T PB2 protein sequences:**

MERIKELRNLMSQSRTREILTKTTVDHMAIIKKYTSGRQEKNPALRMKWMMAMKYPITADKRITEMIPERNEQGQTLWSKMNDAGSDRVMVSPLAVTWWNRNGPVTSTVHYPKIYKTYFEKVERLKHGTFGPVHFRNQVKIRRRVDINPGHADLSAKEAQDVIMEVVFPNEVGARILTSESQLTTTKEKKEELQGCKISPLMVAYMLERELVRKTRFLPVAGGTSSVYIEVLHLTQGTCWEQMYTPGGEARNDDVDQSLIIAARNIVRRATVSADPLASLLEMCHSTQIGGIRMVNILRQNPTEEQAVDICKAAMGLRISSSFSFGGFTFKRTSGSSVKREEEVLTGNLQTLKIRVHEGYEEFTMVGRRATAILRKATRRLIQLIVSGRDEQSIAEAIIVAMVFSQEDCMIKAVRGDLNFVNRANQRLNPMHQLLRHFQKDAKVLFQNWGIESIDNVMGMIGILPDMTPSTEMSMRGVRISKMGVDEYSSAEKIVVSIDRFLRVRDQRGTVLLSPEEISETQGTEKLTITYSSSMMWEINGPESVLVNTYQWIIRNWETVKIQWSQNPTMLYNKMEFEPFQSLVPKAVRGQYSGFVRTLFQQMRDVLGTFDTAQIIKLLPFAAAPPKQSRTQFSSLTINVRGSGMRILVRGNSPVFNYNKTTKRLTVLGKDAGPLTEDPDEGTAGVESAVLRGFLILGKEDRRYGPALSINELSNLAKGEKANVLIGQGDVVLVMKRKRNSSILTDSQTATKRIRMAIN*

**Materials and methods**

**The Mitotoxicity assay**

Serum-free glucose medium: High-sugar DMEM medium containing 25 mM glucose, 1 mM sodium pyruvate, and 5 mM HEPES; Serum-free galactose medium: Sugar-free DMEM medium containing 10 mM galactose, 2 mM glutamine, 1 mM sodium pyruvate, and 5 mM HEPES.

HepG2 cells were suspended with serum-free glucose medium and serum-free galactose medium, respectively and seeded at a density of 2,000 cells per well in 384-well cell culture plates. The cells were then cultured overnight in a 5% CO_2_, 37°C incubator. The next day, diluted compounds were added. Cells were cultured in a 5% CO_2_, 37°C incubator for 24 hours. Each well of cell viability was then measured with the CellTiter-Glo reagent. IC_50_ values were analyzed using GraphPad Prism software for nonlinear fitting analysis of the inhibitory activity of the compounds. IC_50_ (Glucose) / IC_50_ (Galactose) < 3 means no mitotoxicity; 5 > IC_50_ (Glucose) / IC_50_ (Galactose) ≥ 3 means potent mitotoxicity; IC_50_ (Glucose) / IC_50_ (Galactose) ≥ 5 means mitotoxicity.

**Reference**

[1] Chen X, Ma Q, Zhao M, et al. Preclinical Study of ZSP1273, a Potent Antiviral Inhibitor of Cap Binding to the PB2 Subunit of Influenza A Polymerase. Pharm Basel Switz. 2023;16(3):365.
